# Physical Determinants of Fibrinolysis in Single Fibrin Fibers

**DOI:** 10.1371/journal.pone.0116350

**Published:** 2015-02-25

**Authors:** Igal Bucay, E. Tim O’Brien, Steven D. Wulfe, Richard Superfine, Alisa S. Wolberg, Michael R. Falvo, Nathan E. Hudson

**Affiliations:** 1 Department of Physics and Astronomy, University of North Carolina, Chapel Hill, North Carolina, United States of America; 2 Department of Pathology and Laboratory Medicine, UNC School of Medicine, Chapel Hill, North Carolina, United States of America; 3 Program in Cellular and Molecular Medicine, Children’s Hospital Boston, and Department of Biological Chemistry and Molecular Pharmacology, Harvard Medical School, Boston, Massachusetts, United States of America; University Hospital Medical Centre, GERMANY

## Abstract

Fibrin fibers form the structural backbone of blood clots; fibrinolysis is the process in which plasmin digests fibrin fibers, effectively regulating the size and duration of a clot. To understand blood clot dissolution, the influence of clot structure and fiber properties must be separated from the effects of enzyme kinetics and perfusion rates into clots. Using an inverted optical microscope and fluorescently-labeled fibers suspended between micropatterned ridges, we have directly measured the lysis of individual fibrin fibers. We found that during lysis 64 ± 6% of fibers were transected at one point, but 29 ± 3% of fibers increase in length rather than dissolving or being transected. Thrombin and plasmin dose-response experiments showed that the elongation behavior was independent of plasmin concentration, but was instead dependent on the concentration of thrombin used during fiber polymerization, which correlated inversely with fiber diameter. Thinner fibers were more likely to lyse, while fibers greater than 200 ± 30 nm in diameter were more likely to elongate. Because lysis rates were greatly reduced in elongated fibers, we hypothesize that plasmin activity depends on fiber strain. Using polymer physics- and continuum mechanics-based mathematical models, we show that fibers polymerize in a strained state and that thicker fibers lose their prestrain more rapidly than thinner fibers during lysis, which may explain why thick fibers elongate and thin fibers lyse. These results highlight how subtle differences in the diameter and prestrain of fibers could lead to dramatically different lytic susceptibilities.

## Introduction

The hemostatic system must strike a balance between hemorrhage and vessel occlusion. Blood clots must be strong enough to limit extravascular blood flow following injury, yet dissolve appropriately during wound healing [1]. Inappropriate clot formation within blood vessels (thrombosis) leads to ischemia and tissue loss. Blood clots contain an interconnected web of fibrin and blood cells, including platelets, that mediate clot retraction. When the hemostatic role of the clot is fulfilled, it is removed via the fibrinolytic system [2]. The primary enzyme mediating fibrinolysis is the serine protease plasmin, which cleaves the fibrin molecule at specific sites [3]. Plasmin is the activated form of plasminogen and is produced either in the blood stream by urokinase-type plasminogen activator (uPA), or on the fibrin surface, where bound plasmin and tissue-type plasminogen activator (tPA) are protected from their respective inhibitors, α_2_-antiplasmin and plasminogen-activator-inhibitor 1 [3,4].

**Figure 1:**
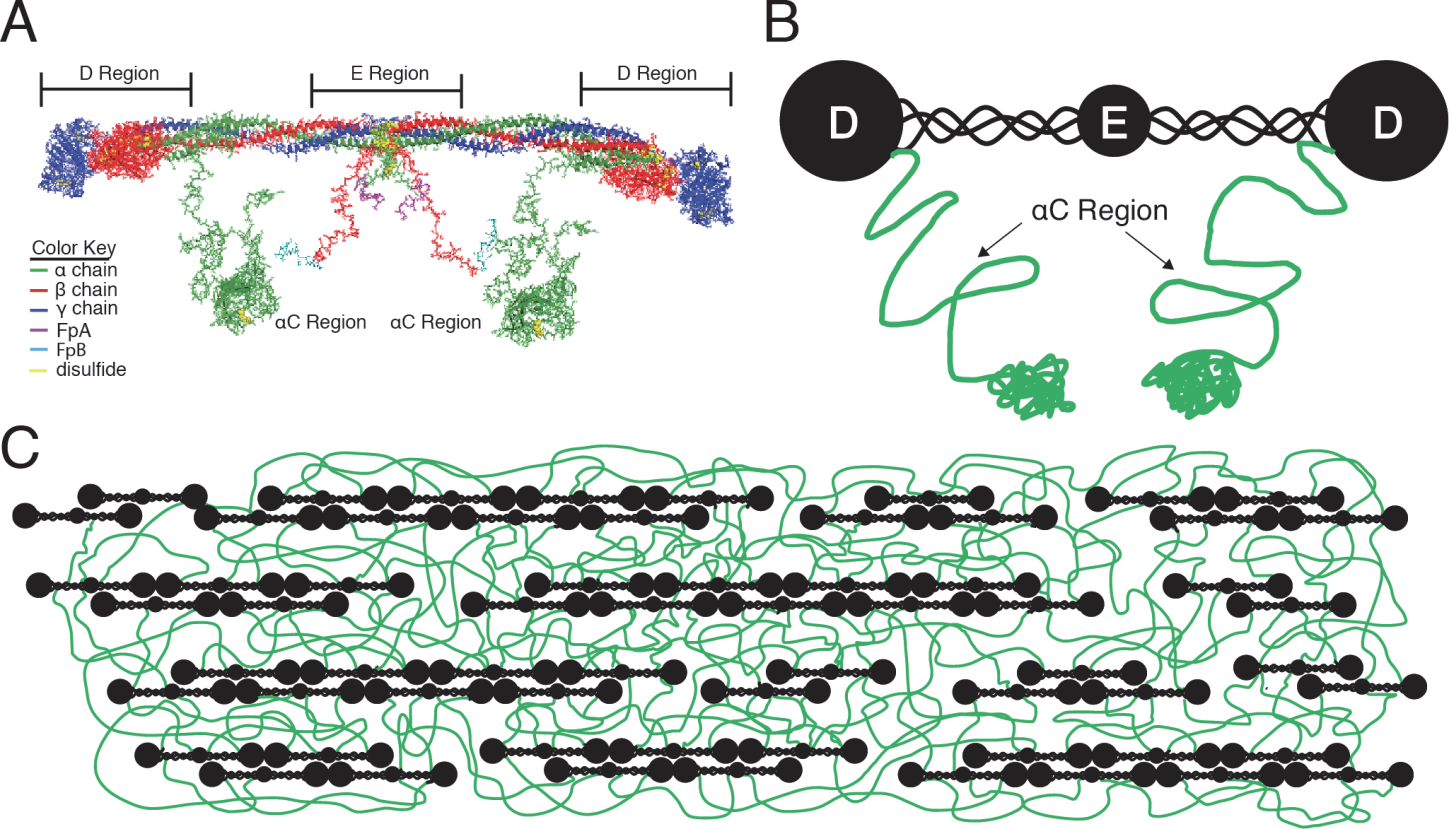
Structure of the Fibrinogen Molecule. **A** structure of the fibrinogen molecule (crystal structure 3GHG) with αC domains built in using homology modeling and Discrete Molecular Dynamics Simulations [24]. Fibrinogen spans 45 nm and consists of two outer D regions, each connected by a coiled-coil segment to the central E region. Thrombin cleaves fibrinopeptides A and B from the Aα-chains and Bβ-chains, respectively, producing insoluble fibrin monomers that form fibrin networks. **B** A cartoon representation of the molecule, with the αC region highlighted in green. **C** A cartoon model of a fibrin fiber emphasizing the interaction of the αC polymer network within the fiber.

The fibrinogen molecule is comprised of three pairs of distinct peptide chains: two Aα-chains, two Bβ-chains, and two γ-chains, which are interlinked by disulfide bridges (Fig. 1) [1,5–7]. Following thrombin-mediated cleavage of fibrinopeptides A and B from the N-termini of the Aα- and Bβ-chains, respectively, fibrin monomers polymerize into half-staggered, double-stranded protofibrils that bundle into fibers via interactions of the αC regions [8]. Cryptic plasminogen and plasmin binding sites in fibrinogen and fibrin monomers [9] are exposed by fibrin monomer polymerization [3]. In particular, the αC regions contain lysine-dependent tPA- and plasminogen-binding sites (K_d_ = 16–33 nM) within residues Aα392–610 [10,11]. During fibrinolysis, plasmin initially cleaves the αC regions, and then cleaves the three polypeptide chains connecting the central (E) and end (D) regions (Fig. 1), which contain low-affinity (K_d_ = 1 μM), lysine-independent plasmin and tPA binding sites [10,12–14]. These cleavages produce COOH-terminal lysine residues in fibrin, which provide additional binding sites for plasmin, and accelerate the dissolution process [10,15].

Understanding clot lysis requires knowledge of enzyme activation kinetics, perfusion rates of enzymes into thrombi, and the effects of structural and physical properties of individual fibrin fibers on the rates of dissolution. The kinetic properties of fibrinolysis depend on the architecture and physical properties of both blood clots (e.g., density, stiffness) and individual fibrin fibers (e.g., diameter, stress) [16–21]. Throughout fibrinolysis, fibrin fibers undergo significant morphological and biochemical changes as thrombi are digested by plasmin [18,21,22]. However, the mechanism of fiber fibrinolysis—whether fibers are transected at one point, or whether the radius of the fiber decreases uniformly—remains unresolved [16,23]. To date, studies of fibrinolysis have occurred at the whole clot level, where it is difficult to resolve the effects of perfusion rates and enzyme kinetics from the effects of individual fiber physical properties and architecture.

Here, we report the progression of fibrinolysis on single fibrin fibers using fluorescent optical microscopy and scanning electron microscopy (SEM). We found that fibers were transected at one point, but the ability of plasmin to cleave fibers depended upon the inherent fiber strain and initial diameter. In particular, we found that when fibers were exposed to plasmin, thin fibers were readily cleaved (lysed), but thicker fibers actually grew in length during fibrinolysis. These results suggest that the lytic susceptibility of a fiber is directly related to the intrinsic strain on the fiber resulting from the polymerization process (its “prestrain”). Based upon the current understanding of fiber architecture and polymerization, we developed continuum mechanics and polymer physics models to determine the relationship between fiber strain and fibrinolysis. We found that as the outer shell of the fiber is lysed, the equilibrium length of the fiber increased, resulting in elongation and loss of fiber prestrain. Thicker fibers elongated at a lower percentage of fibrinolysis than thinner fibers. Our findings suggest fiber prestrain is a prerequisite for lysis: thin fibers are lysed rapidly until prestrain is lost due to fiber transection, whereas thick fibers elongate, reducing prestrain and consequently lytic rates. These data emphasize the importance of the physical properties of individual fibers in determining the fate of blood clots.

## Materials and Methods

### Fibrin Polymerization and Lysis

Structured Surfaces (SSs) were prepared as described previously [24] using a PDMS stamp (Polydimethylsiloxane: Sylgard 184; Dow Corning Corp., Midland, MI, 48686) and urethane glue cured with ultraviolet light (Norland Optical Adhesive 81 (Norland Products, Inc., Cranbury, NC) to make non-extensible, transparent ridges 10 μm high and 20 μm apart on glass coverslips. Fibrinogen (Peak 1 human fibrinogen, Enzyme Research Labs (ERL, Indianapolis, IN, stock solution of 2.37 mg/mL in 20 mM Tris-HCl/ 0.15 M NaCl/ pH 7.4) was thawed from −80°C frozen aliquots at 0.6 mg/mL, diluted in 20 mM HEPES, 150 mM NaCl (HEPES-buffered saline, HBS) to 0.04 mg/mL (~60-fold dilution), and 10 μL placed on a structured surface. Human alpha-thrombin (ERL, stock solution of 3.33 mg/mL at 3000 U/mg = 10000 U/mL in buffer containing 50 mM Sodium Citrate/0.2 M NaCl/0.1% PEG-8000/pH 6.5) was diluted ~5000-fold in 4 mM calcium chloride in HBS to 2X the final needed for each experiment (normally 2.2 U/mL). Equal volumes of thrombin and fibrinogen were then mixed on the SS to give final concentrations of 0.02 mg/mL fibrinogen and 1.1 U/mL thrombin unless otherwise indicated. After mixing, samples were placed into humidified chambers (wet paper in closed petri dish) and placed in a 37°C incubator for thirty minutes to allow polymerization. After polymerization, the reaction buffer was gently pipetted off the sample and HBS without fibrinogen or thrombin was added. For thickness measurements using SEM, a final concentration of 0.1 mg/mL fibrinogen was used to increase the number of fibers. Human plasmin (ERL, stock solution of 2.1 mg/mL at 6 U/mg = 12.6 U/mL in 100 mM Hepes and 100 mM sodium acetate buffer) was thawed quickly and diluted with 4 mM CaCl_2_ HBS buffer to 2X the concentration needed for that sample. Plasmin was then added to the samples at an equal volume to that present, and gentle pipetting was used to thoroughly mix the plasmin with the HBS on the sample. All experiments were performed over a 30-min or 24-hr period, as indicated.

### Fluorescent Microscopy

For samples imaged with fluorescent microscopy, after polymerization but prior to the addition of plasmin, the polymerized fibers were fluorescently labeled by gently exchanging the buffer on the SS with a 1/10,000 dilution of 20 nm thick volume labeled carboxylic acid labeled beads (Molecular Probes, Life Technologies, Inc) in HBS for two minutes, and then the bead suspension was gently removed and replaced with 4 mM calcium HBS. For light microscopy. samples were placed face-up on a Nikon Diaphot 200 microscope with a 100X 1.3 N.A oil objective with epi-fluorescence illumination (Nikon Instruments, Melville, NY)), and appropriate filters, and images recorded with a Cooke PCO 1600 camera and CamWare capture software (Cooke Corporation, Romulus, MI).

### SEM Imaging

The fibrin samples used for SEM imaging were suspended over channels of PDMS and prepared in the same manner, at the same concentrations, and with the batches of protein as the samples used in fluorescence microscopy. Samples were fixed by exchanging the sample buffer with 0.1% glutaraldehyde in HBS and incubated for five minutes. Samples were washed gently with HBS, and then the buffer was exchanged with a graded series of ethanol, critical point-dried sputter coated with 6 nm of gold-palladium, and imaged with a Hitachi S4700 SEM. Single fibrin fibers with no visible branching were selected for measurement, and the thinnest diameter along the length of each of these fibers was recorded. Fibers greater than 1000 nm in diameter were not counted. These were very rare, and appeared to be bundles of smaller fibers.

## Results

### Changes in Fiber Shape during Fibrinolysis

**Figure 2:**
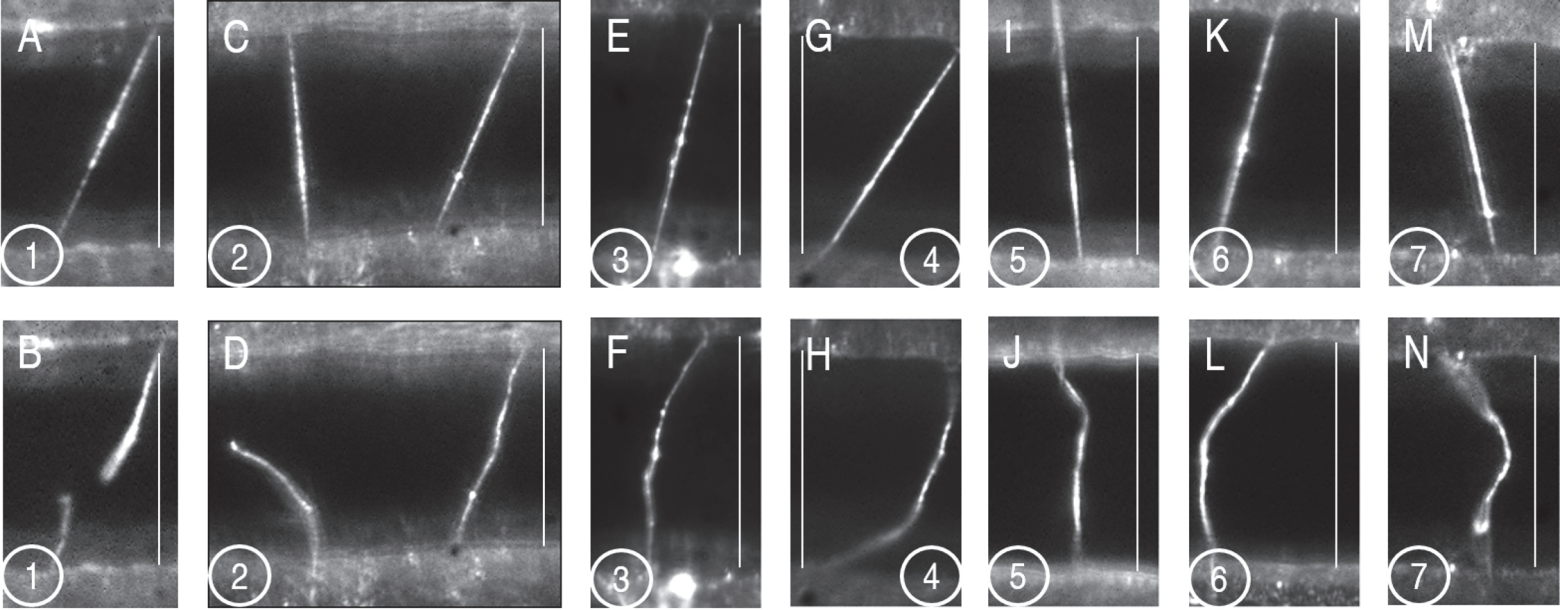
Images of elongated and lysed fibrin fibers. Epi-fluorescence microscopy of fibrin fibers suspended across structured surfaces (bar = 20 μm). Samples were labeled with 20 nm red fluorescent beads and polymerized with 0.11–11 U/mL of human thrombin. Images of fibers before (A, C, E, G, I, K, M) and 5–15 minutes after (B, D, F, H, J, L, N) addition of plasmin. Samples 1–2 were treated with 20 μL of 1.0 U/mL of plasmin and display a lysed fiber (B) or a lysed and elongated fiber in the same field of view (D). Samples 3–7 were treated with 20 μL of plasmin ranging from 0.6 U/mL–6.0 U/mL and show elongated fibers, exhibiting extensions up to 10%.

**Figure 3:**
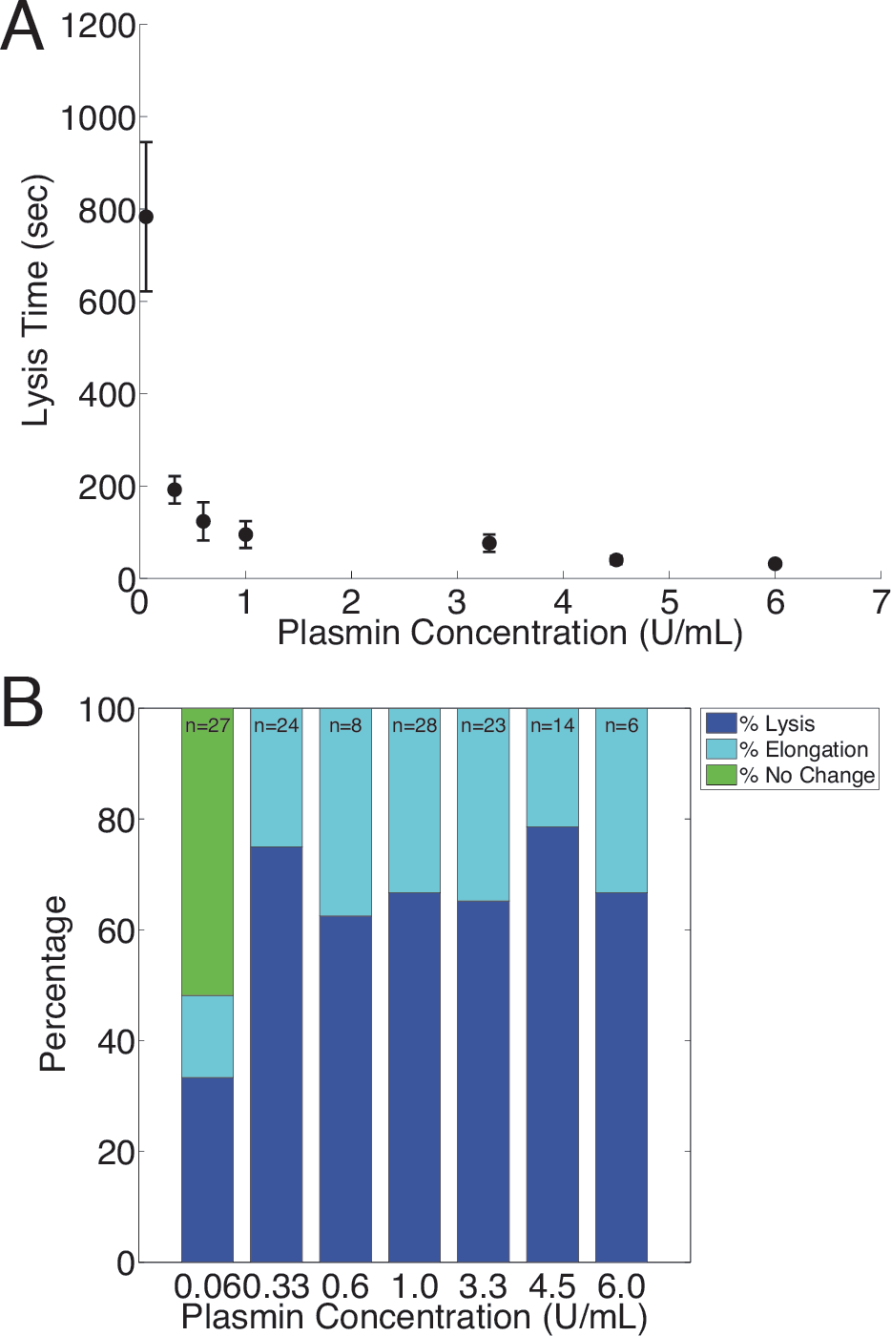
Effect of Plasmin concentration on Lysis and Elongation. **A** The average time measured for single fibers to lyse after exposure to plasmin. Fibers exhibiting elongation were not counted in this analysis. **B** Percentage of fibers that exhibited lysis, elongation, and no change within thirty minutes after exposure to plasmin. Note that the ratio of the number of fibers that elongated to that of fibers that lysed remained constant across the range of plasmin concentrations. ‘n’ indicates the total number of fibers (lysed, elongated, no change) observed per plasmin dose.

To study individual fiber behavior during fibrinolysis and the timescales within which plasmin can lyse individual fibers, we polymerized and suspended fibrin across micropatterned ridge-and-valley structures, as previously described [25]. Fibrin fibers were labeled with 20 nm red fluorescent beads and observed under an optical microscope. We then added plasmin (0.06–6 U/mL) to the fibrin samples and recorded fiber behavior for thirty minutes. Of fibers that lysed, fibers treated with the lowest plasmin concentration (0.06 U/mL) lysed within 13 minutes on average and all other plasmin concentrations resulted in more rapid fiber lysis (Figs. 2A–D and 3A).

Fibrin fibers displayed two distinct conformational changes after being treated by plasmin: complete lysis (in which fibers were cleaved transversely at a single point) or elongation (in which fiber length increased, resulting in a decrease in apparent tautness and fiber prestrain) (Fig. 2, S1–S3 Movies). Elongation and complete lysis (different fibers) were frequently observed within the same sample, independent of plasmin concentration (Fig. 2C–D). We found that 64 ± 6% of fibers lysed transversely at a single point, resulting in two segments of the original fiber with one side of each segment still attached to the structured surface. Interestingly, after the initial lysis event, the fibers were not degraded further during the thirty-minute period of observation. These findings suggest that once the fiber is initially cut, the binding sites for further binding/cleaving become less accessible to plasmin.

Surprisingly, at least 29 ± 3% of fibers remained intact and suspended across the ridges of the structured surfaces; these fibers exhibited an increase in length from their original length (Fig. 2A–J). As these “elongated” fibers extended, they underwent additional morphological changes resulting in a loose, curved configuration exhibiting thermal vacillations that were nonexistent in taut fibers not exposed to plasmin (see S1–S3 Movies). Because fibers are known to form in a prestrained, tensed state [26,27], these structural changes imply that plasmin cleavage decreases the inherent tension of the fiber.

We performed control experiments to determine whether elongated fibers were in a transient, partially lysed state. First, we added 3.3 U/mL plasmin and allowed elongation to occur for one hour. We then added an additional high dose of plasmin (6.0 U/mL) while observing the already elongated fibers; in these experiments, we did not observe transverse cleavage or additional extension of the fibers, suggesting that elongation is not a result of low plasmin activity, but rater, elongated fibers are in a state where the lytic susceptibility of the fiber is greatly reduced. Second, we tested the hypothesis that fibrinolytic rates are reduced in elongated fibers, but eventually the fibers will lyse. For this experiment, we observed several samples after 24 hours of exposure to 3.3 U/mL plasmin; fiber fragments of lysed fibers were still present and elongated fibers remained in the same state without transverse cleavage or additional lengthening. These results indicate that fibrin fibers can either be cleaved at one point (i.e., lyse), or elongate. The elongated state of the fiber is not a transitional state on the pathway to lysis, but a fundamentally different state, without inherent strain, with a greatly reduced lytic susceptibility.

### Effects of Plasmin Concentration on Fibrinolysis Rate and Elongation

To determine the effect of plasmin concentration on fiber lysis time, individual fibrin fibers were polymerized on structured surfaces using a fixed thrombin concentration (1.1 U/mL) and then exposed to 0.06, 0.33, 0.6, 1.0, 3.3, 4.5, or 6.0 U/mL plasmin. Fibers were imaged for thirty minutes, and the lysis time (the time required for a fiber to fully lyse after exposure to plasmin) was recorded and plotted (Fig. 3A). Fibrin samples were sorted into three groups: those that lysed, those that elongated, and those that demonstrated no visible change in physical structure (Fig. 3B).

For fibers that lysed, the average time measured for single fiber lysis at various plasmin concentrations is displayed in Fig. 3A. For plasmin concentrations above 0.06 U/mL, the ratio of fibers exhibiting fibrinolysis vs. elongation shows now plasmin concentration dependence (Fig. 3B). These data suggest that elongation is not a result of low plasmin activity or concentration, and that the determinants of whether a fiber lyses or elongates relate to the physical properties of individual fibers rather than enzyme kinetics. Two candidate physical parameters are fiber diameter and fiber prestrain.

### Effect of Fiber Diameter on Elongation

**Figure 4:**
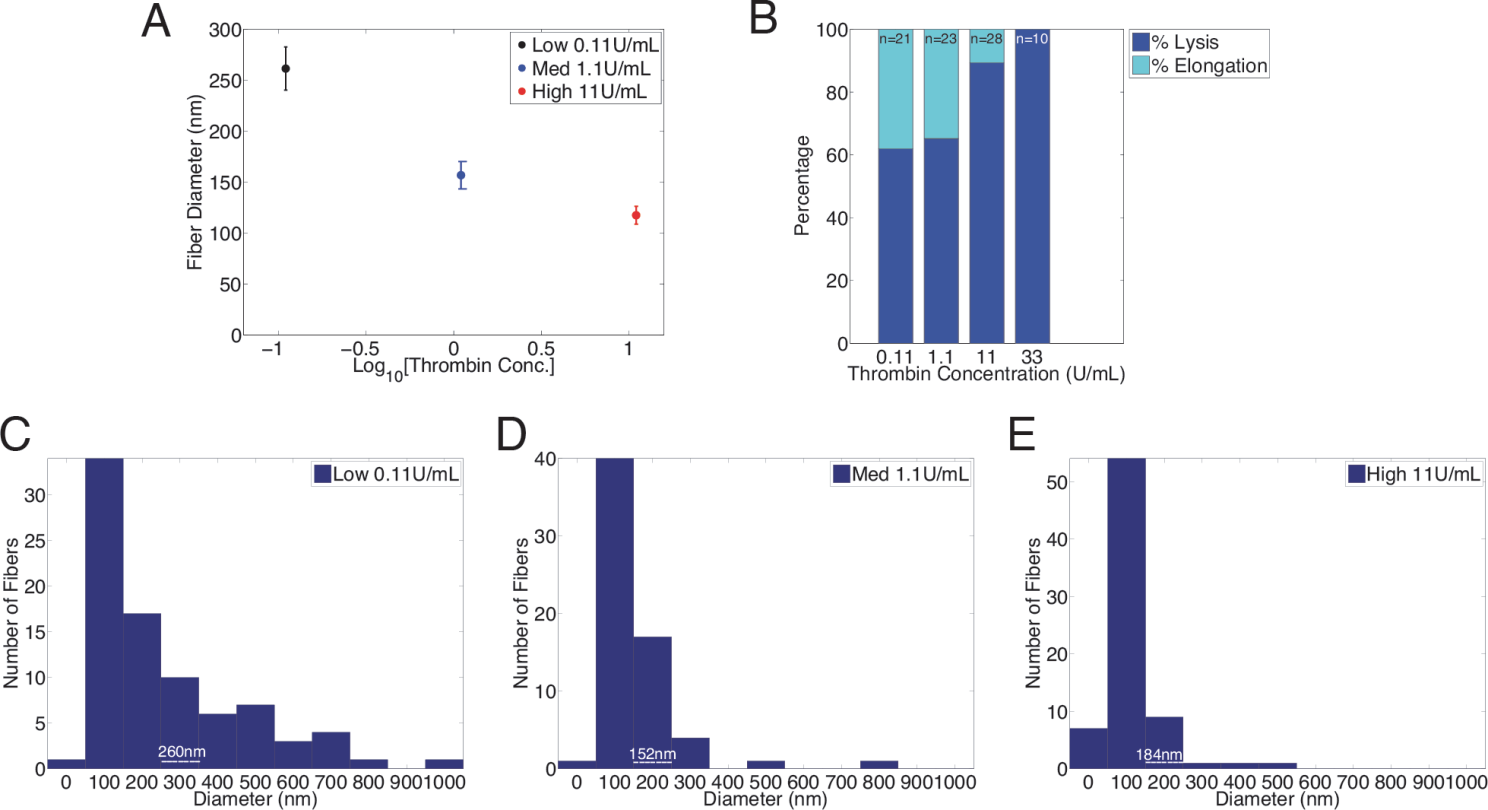
Diameter Dependence of Fibrinolysis. **A** Over two hundred single fibers were imaged on the SEM. Fiber diameters were measured at the thinnest point. Data were collected from fibers polymerized at three distinct thrombin concentrations: 0.11 U/mL (84 samples), 1.1 U/mL (64 samples), and 11 U/mL (73 samples), (p < 0.02 for all cases). **B** Percentage of fibers that lysed or elongated during the first thirty minutes of exposure to 3.3 U/mL of plasmin. Fibrin fibers polymerized by higher concentrations of thrombin lysed more frequently than those polymerized by lower thrombin concentrations. **C–E** The data in the histograms were segregated according to the percentage of fibers that lysed or elongated. The percentage of fibers with thicknesses below the *d*_0_ diameters (indicated by a white dotted line) parallels the percentage of fibers that lysed in the corresponding bar graphs. The threshold diameter (*d_0_*) was 200 nm ± 30 nm.

Previous work has indicated that fibrin diameter is inversely proportional to thrombin concentration [1,17,23,28–30]. To confirm these findings, fibrin samples polymerized by three different thrombin concentrations (0.11, 1.1, and 11 U/mL) were imaged using scanning electron microscopy (SEM); the average fiber diameter for each concentration is displayed in Fig. 4A. Consistent with the earlier reports, our data indicate that fiber diameter decreases with increasing thrombin concentration [29].

Next, samples of fibrin fibers were prepared on structured surfaces for fluorescent imaging using 0.11, 1.1, 11, and 33 U/mL thrombin. Samples were then treated with 3.3 U/mL of plasmin (a concentration that yielded a mixed distribution of lysed and unlysed [elongated] fibers, Fig. 3), observed by optical microscopy, and the number of fibers that elongated or lysed was recorded (Fig. 4B). Fibers polymerized with lower thrombin concentrations were more susceptible to elongation, suggesting thicker fibers are more likely to elongate. We hypothesized that there is a threshold diameter, below which fibers lyse, and above which fibers elongate. To estimate this threshold, the fiber diameter histograms (Fig. 4C–E) were segregated according to the elongation percentages found for each thrombin concentrations in Fig. 4B. For example, since 89% of fibers polymerized by 1.1 U/mL thrombin lysed, we estimated the threshold fiber diameter to be 184 nm because 89% of fibers had smaller diameters than 184 nm, while 11% of fibers were thicker than 184 nm. Averaging the threshold diameter found for each thrombin concentration yielded a threshold diameter of 200 ± 30 nm separating the lysed and elongated fibers. These findings indicate that a fiber’s susceptibility to elongation is largely determined by its diameter—the probability of elongation is reduced if the fiber diameter is below the threshold.

### Mathematical Modeling of Fiber Elongation

The observation that fibrinolytic activity was reduced both after fibers are initially cleaved and in elongated fibers that were never fully cleaved suggests the ability of plasmin to cleave fibrin is directly related to the mechanical state of the fiber. Although our prior work and other investigations show that fibers are under tension after the polymerization process [21,22,31], the random curvilinear conformations of the fibrin fibers (Fig. 2A–J) and the thermal fluctuations observed after fibrin lysis (Fig. 2K, L) indicated that fibers were under little or no tension after lysis or elongation. We therefore hypothesized that plasmin’s enzymatic activity depends on fibrin fiber tension. In the case of elongated fibers, the simultaneous loss of tension and increase in length suggests the fiber undergoes a fundamental change in its equilibrium mechanical state. The equilibrium length of the fiber prior to lysis, or its initial length (*L_i_*) is less than the inter-ridge (structured surface) distance, *L_SS_*, while after lysis, the final equilibrium length of the fiber, *L_F_*, is longer than the inter-ridge distance.

$$L_{F}>L_{SS}>L_{i}$$

Within this picture, the fiber in its native, pre-lysis state as it spans the micro-patterned ridges is in a state of tension (or prestrain). Our data suggest that the ability of plasmin to effectively bind and engage enzymatic activity requires a certain threshold fiber strain, ε^*^ (*ε* = (*L_SS_-L_i_*)/*L_i_* ). If this prestrain is lost before the fiber completely lyses or after the initial cleavage event, then lytic activity effectively ceases. We developed two separate mathematical models based upon the structure of the fibrin fiber to test these hypotheses. Our models provide an explanation for how polymerization produces a prestrain in the fiber that is then lost during the fibrinolytic process, which in turn, leads to fiber elongation (both models are described in detail in S1 File).

### Polymer Network Model

We, and others, previously observed that the fibrin fiber itself behaves as a polymer network; the unstructured αC domains of the fibrin molecules form a network within the fiber through intermolecular interactions [32,33]. As a polymer network, the equilibrium length of the fibrin fiber depends on the degree of intermolecular links between the αC regions. The Flory-Rehner equation [26,34] relates polymer swelling to the degree of interlining of polymer chains within the network:

$$Q\propto \rho_{c}^{-3/5}$$

where *Q* is the swelling ratio or the ratio of the network volume (polymer and solvent) relative to the dry polymer volume, and *ñ_c_* is the density of crosslinks (equivalent to the number of polymer segments per unit volume contributing mechanically to the network [34,35]). This equation predicts that a polymer network will decrease in volume as the number of crosslinks between polymer strands increases. The final step of fibrin polymerization is the association of αC domains into networks termed α-polymers. We hypothesize that these interactions are analogous to crosslinks in a polymer gel and will lead to a shrinking of fiber volume and equilibrium length, resulting in fiber prestrain (see S1 File and S1 Fig.). Accordingly, the Flory-Rehner equation predicts that a fiber will swell as the number of crosslinks within the network is reduced (which will occur during lysis). In this way, the αC polymer network model for fibrin fibers provides a mechanism to account for fiber prestrain and elongation during lysis.

### Continuum Mechanics Model

**Figure 5:**
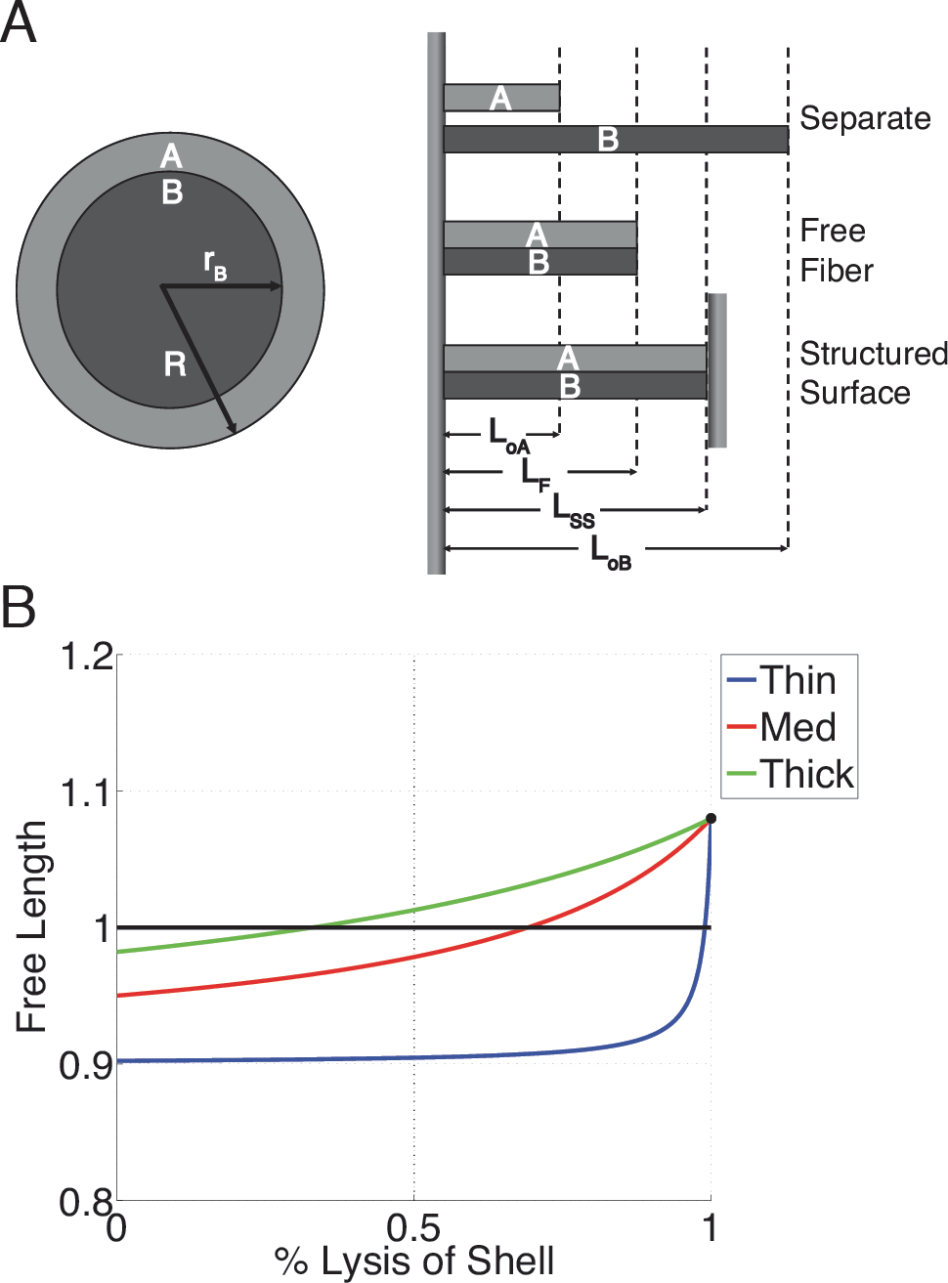
Modeling the lysis of a Fiber Consisting of an Inner Core and Outer Shell. **A** Left: cross-section of a fiber with an outer shell (A) and inner core (B). Right: a portrayal of the relative lengths of the shell, the core, the free fiber (equilibrium fiber length), and SS-suspended fiber. **B** The fiber free length, normalized to the length between the structured surfaces, for thin (blue, 40 nm radius), medium (red, 80 nm radius), and thick (green, 120 nm radius) fibers as the shell is lysed. The black line signifies the SS length (*L_SS_*) and the black dot is the free length of the fiber after 100% lysis of the shell (i.e., the core length). Note that there is no radial dependence on the free length for fibers of the constant ratio model. For this plot *L_oA_* = 18 μm, *S* = 1.2. For the constant core, the core thickness was 15 nm.

Fibrin fibers have a well-defined 23 nm banding pattern when viewed by electron microscopy [36]. The banding has been ascribed to protofibrils wrapping outwardly around the center of the fiber and in registry with the inner protofibrils; for this to be true, the outer protofibrils must be stretched to align with the inner molecules [21,27]. Based on this picture, we propose a simplified core-shell model with two mechanical “domains” with the same elastic modulus *E*: domain A (outer shell, which is stretched) and domain B (inner core, which is compressed) (Fig. 5A). In a taut fiber suspended across a structured surface (SS) or within a fibrin clot network, the shell is stretched and the core is compressed. The free fiber length *L_F_* is the equilibrium length of the conjoined domains and the requirement that a fiber be taut pre-fibrinolysis necessitates the condition that *L_F_ < L_SS_*. *L_F_*, will therefore change as the outer shell is lysed as given by:

$$L_{F}^{'}=SL_{oA}\left(\frac{(1-x)R^{2}+xr_{B}^{2}}{S(1-x)(R^{2}-r_{B}^{2})+r_{B}^{2}}\right)$$

Where *R* is the fiber radius, *r_B_* is the core radius, x is the percentage of the outer shell lysed, and we have imposed the constraint that *L_oB_ = SL_oA_*, where *S > 1* is a proportionality constant relating *L_oB_* and *L_oA_*, and *L_oB_* and *L_oA_* are the initial length of the core and shell respectively (Fig. 5A). In this model, if fibrin fibers of all diameters share a common outer shell thickness, then thicker fibers elongate at a much earlier stage of fibrinolysis than thinner fibers, in agreement with our experimental data (Fig. 5B). Two other models of fibrin architecture (constant core radius, and constant core:shell radius ratio) do not show the correct increase in length as the fiber lyses (see S1 File and S2 Fig.). Together, our experimental and modeling data indicate that fiber diameter determines the prestrain induced by fibrin polymerization, and the pre-strain, in part, governs a fiber’s susceptibility to plasmin. Combining our models involving fiber strain during lysis, with other recently published models of lysis involving stochastic reactions and enzyme diffusion may be required for a complete picture of fibrinolysis [37,38].

## Discussion

Understanding fibrinolytic mechanisms requires a detailed understanding of lysis on both the scale of whole clots and that of individual fibers. Most previous studies have evaluated whole clot lysis, while knowledge of lytic mechanisms on the scale of individual fibers is limited. Studying the lysis of isolated fibers is complementary to previous confocal and electron microscopy studies of fiber lysis within networks [16,17,20] and may enable the identification of phenomena that would be otherwise obscured by network effects. Our investigation of individual fiber lysis produced three important findings. First, we observed that some fibers elongate, rather than lyse, in response to fibrinolytic activity, revealing heterogeneity in the fibrinolytic susceptibility of clots on the scale of individual fibers. Second, for fibers that elongated, lytic activity was greatly reduced or eliminated even when exposed to high plasmin concentrations. This suggests that elongating fibers transition from a state of high tension and prestrain to a state of low or no tension or strain, and that the ability of plasmin to bind fibrin and lyse fibers is strain-dependent (i.e., a certain threshold strain, ε^*^, is necessary for lysis). Third, fiber diameter determined fiber fate; thicker fibers were more likely to elongate, whereas thinner fibers were more likely to lyse, suggesting larger fibers fall below the threshold strain when still partially lysed, whereas thinner fibers maintain strain above ε and facilitate lysis. Together, these novel findings reveal that clot lysis is regulated not only by global mechanisms (e.g., diffusion of fibrinolytic enzymes into the clot), but also by biomechanical functions on the level of individual fibers.

Our mathematical modeling provides a theoretical framework for understanding fiber prestrain and elongation, and further experimental and modeling work should attempt to determine the exact value of ε^*^. Both the polymer network model and the two-state elastic solid model predict that the fiber will begin in a tensed, prestrained state that will relax and expand during lysis. The continuum mechanics model illustrates how the lysis of the outer protofibrils of a fiber can lead to elongation, and shows how thicker fibers lose prestrain much more rapidly than thinner ones, but does not predict a mechanism by which the unstrained core can be lysed. The polymer physics model provides a mechanism for elongation and the timescales of lysis, but does not directly account for the diameter dependence of elongation. Future modeling could combine the continuum mechanics approach with the polymeric properties of fibrin to capture the full strain and diameter dependence of lysis. This combined model should include plasmin kinetics and realistic molecular binding sites and accurate molecular packing geometries within the fiber.

Our models for fiber elongation correspond with known data regarding the molecular binding sites of plasmin and tPA on fibrin, and their exposure during polymerization. Previous studies have revealed two pairs of binding sites for tPA and plasm(ogen): one pair resides in the D region with a tPA site at γ312–324 and a plasminogen site at Aα148–160, and the second pair resides on the αC region [10]. All are cryptic in fibrinogen as well as in monomeric fibrin, but are made accessible with polymerization [9–11,39]. The mechanism of exposure of the sites may be related to a conformational change in the structure of D region during polymerization [39]. Likewise, recent investigations demonstrate that polymerization, and more specifically inter-molecular αC-αC interactions, are necessary to expose the tPA and plasminogen sites on the αC region [40]. Given that lysis begins in the αC region [10] followed by cleavage in the D region, conditions favorable for lysis of the αC region (exposure of the αC sites) may be a necessary step in exposing the D region sites.

One potential molecular mechanism consistent with our data as well as prior studies on tPA and plasminogen binding sites, is stretching of the αC regions. Global fiber prestrain in the as-polymerized fiber implies local molecular scale strain, which we propose is accommodated by the stretching of the αC regions of the molecule. The origins of the prestrain may be related to the formation of the αC-αC network that forms through their association during polymerization. The Flory-Rehner equation predicts an equilibrium volume reduction when crosslink density increases (in this case αC-αC associations) [26]. Since the fiber length is fixed however, the inward-directed entropic forces act to stretch some of the αC regions beyond their equilibrium length (see S1 File). When lysis begins and strain is reduced, as in the case of the larger diameter fibers in our study, the αC regions themselves are relieved of strain or are partially cleaved. The αC regions then coil up leading to the potential encryption of both the αC binding sites for tPA and plasminogen as well as those in the D domain. Therefore, the presence of strain can account for binding site accessibility in polymeric fibrin. This description provides a molecular mechanism for our hypothesis that polymerized fibrin must have an internal strain greater than ε^*^ in order to be laterally transected. Fiber strain can be affected by cross-linking of the αC domains and γ-chains by FXIII [32]; some studies have suggested differential crosslinking in clots composed of fibers with different thicknesses [21,41]. The fibrinogen preparation was not explicitly depleted of factor XIII; therefore, differential fibrin crosslinking in thinner versus thicker fibers may contribute to the observed differences in elongation and stability. Determining how the extent of fiber cross-linking determines lysis times of individual fibers or whether more cross-linking in thicker fibers could promote elongation are interesting areas of research for future studies.

The details of how plasmin lyses fibrin fibers have previously been debated. Our observation that fibers are cleaved transversely agrees with previous studies of fibrin network digestion [16,17]; these data taken using confocal microscopy showed that in both intrinsic and extrinsic fibrinolysis, fibers are transected at a single point before completely dissolving or bundling with other fibers. These findings do not necessarily contradict the radially symmetric progression of fibrinolysis proposed by Diamond et. al. [18], in which the fiber diameter slowly decreases until the entire fiber is digested. Since plasmin binding and cleavage are not limited to a single point on the fibrin surface, it is likely that the initial stages of fibrinolysis do progress radially with the entire fiber diameter decreasing [23,42]. However, our studies of the fibrinolysis of individual fibers show that fibers are cut through at only one point, and after the initial cleavage event, further digestion by plasmin is greatly reduced.

Our data showing that fiber elongation reduces fiber lysis, must be reconciled with the fact that blood clots and fibrin networks do, in-fact, lyse. The strain-dependence of lysis helps to reconcile these apparently contradictory results. Because of the interconnected fiber architecture of a clot, the transection of one fiber necessarily means that the stress within the network will re-equilibrate. Consequently, fibers within a lysing network are constantly subjected to dynamic stresses and strains as fibers around them lyse. The dynamic nature of lysis likely allows thicker fibers to maintain the ε^*^ required for lysis to occur. Additionally, fibers that are cleaved have been observed to aggregate, forming thicker fibers, which may increase strain and enhance lytic rates [16]. It is also interesting to note that following fibrin formation, interactions between fibrin and activated platelets trigger clot retraction, during which fibrin fibers are stretched to the point of stiffening. It has been hypothesized that platelet-induced strain on fibers during clot retraction facilitates lysis [43,44]. Other studies have found that excessive strain hinders fibrinolysis via the expulsion of water and enzymes from the fibrin network due to unfolding of individual monomers and subsequent exposure of hydrophobic domains [19]. We hypothesize that clot retraction keeps fibers in a state above ε^*^, allowing fiber lysis rather than elongation to occur. Future work should directly measure the dependence of lysis/elongation rates on fiber strain to distinguish strain from the effects of enzyme perfusion and activation rates.

Plasma clots from patients with bleeding diathesis, such as hemophilia, contain thicker fibers and are more susceptible to lysis, while clots formed from plasmas of patients with a history of thrombosis contain thinner fibers and exhibit reduced fibrinolysis [45]. Thus, findings from the present work as well as that of others [16] indicating that thinner fibers lyse more rapidly than thicker fibers is unexpected and paradoxical. Moreover, we find that some thicker fibers, in the absence of external stress, are never fully laterally transected. These paradoxes may be reconciled with the fact that lysis of whole clots reflects not only the susceptibility of individual fibers to lysis, but also mechanisms that localize fibrinolytic enzymes within the clot and dynamic architectural rearrangements causing rapidly changing stresses and strains on individual fibers. Fibrinolytic enzymes are likely to perfuse more slowly into dense clots composed of thin, closely-packed fibers than coarse clots composed of thick, loosely-packed fibers. Additionally, dynamic stress redistribution amidst fibers in a lysing clot likely keeps fibers under constant strain. Therefore, our work interrogating fibrinolysis on the scale of individual fibers reveals that complex regulatory mechanisms govern fibrin network biophysical stability, and consequently, the biochemical process of fibrinolysis.

## Conclusion

We have identified a relationship between a fibrin fiber’s susceptibility to lysis and its biomechanical state. Fibers either lyse transversely or elongate depending upon their initial diameter, with thicker fibers being more likely to elongate. Fibers that are transected at one location show a dramatic decrease in the rate of further degradation. Both of these observations indicate that lysis kinetics depend strongly on the inherent tension/strain in the fiber. We find that fibers must be stretched from their equilibrium length beyond a certain threshold strain, or lytic enzymatic activity is greatly reduced. Studying lysis at the individual fiber level provides a quantitative understanding of mechanisms by which fibers are digested and the enzymatic cleavage rate, which in turn, will improve models of clot lysis. Having a complete hierarchical model of fibrinolysis could enable the next generation of thrombolytic therapeutics.

## Supporting Information

S1 File Supporting Information. Polymer physics and continuum mechanics models of fibrin fiber elongation.
(DOCX)

S1 Fig Cartoon model of fibers as αC polymer networks showing the difference between the mechanical equilibrium of a fiber constrained at two ends vs. protofibrils unconstrained at two ends. **A** A structural diagram for the fibrin fiber, consisting of protofibrils (black) with un-crosslinked αC regions (blue), suspended across the structured surfaces (1) and in free space (2). **B** As the unstructured αC regions become intercross linked (red), the network contracts, but the fiber constrained at both ends can only shrink in the diameter, not length-wise. This leads to the fiber being in a pre-strained state; the fiber is stretched beyond its equilibrium length. **C** The number of crosslinks is reduced during fibrinolysis in fibers with sufficient prestrain to undergo binding with fibrinolytic agents. The reduction in the number of crosslinks leads to an expansion of the network and the lysed αC regions form bundles.
(TIF)

S2 Fig Modeling lysis as a function of fiber radius. The fiber free length, normalized to the length between the structured surfaces, for thin (blue, 40 nm radius), medium (red, 80 nm radius), and thick (green, 120 nm radius) fibers as the shell is lysed. The black line signifies the SS length (*L_SS_*) and the black dot is the free length of the fiber after 100% lysis of the shell (i.e. the core length). Note that there is no radial dependence on the free length for fibers of the constant ratio model. For this plot *L_oA_* = 18 μm, *S* = 1.2. For the constant core, the core thickness was 15 nm; for the constant shell, the shell thickness was 35 nm; for the constant ratio, the radius of the core was *half* the radius of the whole fiber.
(TIF)

S1 Movie An elongating fiber treated with 6.0 U/mL of plasmin. The video was sped-up 4x; the time-stamp represents real-time.
(AVI)

S2 Movie An elongating fiber treated with 0.6 U/mL of plasmin. The video was sped-up 6x; the time-stamp represents real-time.
(AVI)

S3 Movie An elongating fiber treated with 1.0 U/mL of plasmin. The video was sped-up 6x; the time-stamp represents real-time.
(AVI)
