# Supplementary material for: Physical Determinants of Fibrinolysis in Single Fibrin Fibers
Source: PLoS One. 2015 Feb 25;10(2):e0116350. doi: 10.1371/journal.pone.0116350 (PMC4340865; doi:10.1371/journal.pone.0116350)
Supplement: S1 File — Polymer physics and continuum mechanics models of fibrin fiber elongation. (DOCX) [file pone.0116350.s001.docx]

Physical Determinants of Fibrinolysis in Single Fibrin Fibers

*Igal Bucay, E. Tim O’Brien III, Steven D. Wulfe, Richard Superfine, Michael R. Falvo, Nathan E. Hudson*

**Modeling Fiber Elongation**

Here, we consider two distinct models of the mechanical properties of the fiber within the dynamic context of lysis. First, we consider the fiber itself as polymer network whose mechanical properties are mediated by entropic elasticity as in a polymer gel [[1](#_ENREF_1),[2](#_ENREF_2)]. In the second approach we consider a two-domain core-shell continuum mechanics model for the fiber, with the outer shell under tensile strain [[3](#_ENREF_3),[4](#_ENREF_4)]. We note that these models are not mutually exclusive but complementary. The polymer model provides a molecular scale mechanistic picture of how lytic activity leads to expansion and elongation, while the continuum model with its core-shell picture of the fiber cross section helps provide a potential explanation for why we see differing elongation behavior for small vs. large fibers.

**αC polymer network model**

We, and others, previously proposed a structural/mechanical model of the fibrin fiber that treats the fibrin fiber itself as a polymer network: the unstructured αC domains of the fibrin molecules form a network within the fiber through intermolecular interactions [[2](#_ENREF_2),[5](#_ENREF_5)]. This is consistent with the fact that αC regions interact to form αC polymers both as isolated fragments, but also within the fibrin fibers themselves [[6-9](#_ENREF_6)]. Our view is that these αC polymers act as a continuous polymer network that is a mechanical background matrix within which the structured protofibrils are imbedded (See Figure S1). The mechanical properties of the fiber in the small strain limit are determined primarily by this polymer network much like in a conventional elastomer (rubber) [[2](#_ENREF_2),[5](#_ENREF_5),[10](#_ENREF_10)].

Viewed as a polymer network, the mechanical properties of the fiber, and in particular its response to strain imposed on the network, is a function of the properties of the individual polymer segments making up the network (the αC regions) and the number of the those segments per unit volume within the fiber. The equilibrium length of the fibrin fiber will depend on the degree of intermolecular links between αC. The Flory-Rehner equation [[11](#_ENREF_11),[12](#_ENREF_12)] relates polymer swelling to the degree of interlining of polymer chains within the network:

$Q\propto\rho_{c}^{-3/5}$ (S1)

where *Q* is the swelling ratio or the ratio of the network volume (polymer and solvent) relative to the dry polymer volume, and *ρ_c_* is the density of crosslinks (equivalent to the number of polymer segments per unit volume contributing mechanically to the network [[11](#_ENREF_11),[13](#_ENREF_13)]). Within a fibrin fiber, the αC polymers are the first targets of lysis [[14](#_ENREF_14)]; therefore, as segments are lysed by plasmin they are effectively removed from the mechanical network and the network expands (See Figure SI1C). The αC polymer network model therefor predicts elongation of fibers that undergo partial lysis and is consistent with our observations for larger diameter fibers.

**Continuum Mechanics Model**

We simplified the fiber into two “domains” with the same elastic modulus *E*: domain A (outer shell, which is stretched) and domain B (inner core, which is compressed) (Figure 5A, main text). In a taut fiber suspended across a structured surface (SS) or within a fibrin clot network, the shell is stretched and the core is compressed, giving rise to the prestrain (the fiber equilibrium length is shorter than its end-to-end distance). The free fiber length *L_F_* is the equilibrium length of the conjoined domains. The initial fiber prestrain means that *L_F_ < L_SS_*. The free fiber equilibrium condition (the internal forces must balance) yields the following relations:

$EA_{A}\varepsilon_{A} + EA_{B}\varepsilon_{B}=0$ (S2)

where *E* is the elastic modulus, $\varepsilon_{A}=(L_{F}-L_{oA})/L_{oA}$ is the shell strain in a free fiber, $\varepsilon_{B}=(L_{F}-L_{oB})/L_{oB}<0$ is the core strain in a free fiber, $A_{A}=\pi(R^{2}-r_{B}^{2})$ is the shell cross-sectional area, $A_{B}=\pi r_{B}^{2}$ is the core cross-sectional area, *R* is the fiber radius, and $r_{B}$ is the core radius. The compressed core has a larger equilibrium length than the stretch shell, thus *L_oB_ = SL_oA_*, where *S >* 1. Substituting into Eqn. S2, the free fiber length is given by

$L_{F}=SL_{oA}\left(\frac{R^{2}}{S(R^{2}-r_{B}^{2})+r_{B}^{2}}\right)$. (S3)

During lysis, the outer shell is cut first, leading to a decrease in outer shell thickness; this releases compression on the inner core and leads to fiber elongation. Here, we assume that the fiber is cleaved radially, with the radius of the fiber decreasing uniformly across the length of the fiber. The equilibrium fiber length $L_{F}^{'}$ as a function of cleavage of the shell is given by:

$L_{F}^{'}=SL_{oA}\left(\frac{(1-x)R^{2}+xr_{B}^{2}}{S(1-x)(R^{2}-r_{B}^{2})+r_{B}^{2}}\right)$, (S4)

where *x* represents the percentage of the outer shell that has been lysed. The core length (*L_oB_*) is thus recovered at *x =* 1 when the outer shell is 100% lysed. In the case when $L_{F}^{'}\geq L_{SS}$, we consider this to be equivalent to the elongated state observed in our experiments.

We considered three independent models for fiber structure: (i) constant shell thickness: $R-r_{B}$ = const., (ii) constant core thickness: $r_{B}$ = const., and (iii) constant ratio model: $R/r_{B}$ = const. > 1. Under each model, we compared the resultant free length $L_{F}^{'}$ for fibers of thin (blue), medium (red), and thick (green) radii as the shell lysed up to 100%, at which point the core length is recovered. During shell lysis, $L_{F}^{'}$ eventually exceeds $L_{SS}$ , which is the definition of “elongation”.

For fibers of equal shell thicknesses (Figure S2, top), it can be seen that thicker fibers elongated at a lower percentage of shell lysis than thinner fibers. This result is in agreement with experimental data (Main Text, Figure 4), which indicates that fibers exhibiting elongation are thicker on average. On the contrary, fibers of equal core thickness (Figure S2, middle) display the opposite: thinner fibers reach the condition for elongation at a lower percentage of shell lysis than thicker fibers. Likewise, fibers adhering to the constant radial ratio model (Figure S2, bottom) are also inconsistent with experimental data: their free length exhibit no dependence on fiber radius.

These three different models predict a difference in fiber prestrain ($\varepsilon_{0}=(L_{SS}-L_{F})/L_{F}$) for fibers of different thicknesses. For fibers of equal shell thickness, thicker fibers (green) have a lower initial fiber strain than thinner fibers (blue), but fibers with constant core thickness exhibit the opposite effect, with thinner fibers having a higher prestrain. Fibers with constant radial ratio have a unitary spread in fiber strain.

These calculations disregard any changes in radius that a fiber may experience or any change in fibrinolysis rate due to a change in length through fibrinolysis, as well as different relative core and shell lengths for fibers of different thicknesses. The approach in these models is also an oversimplification of fibrin fibers, which are naturally not rigidly divided into two distinct domains.

The results from our continuum mechanics models of the fiber show that if the fiber consists of a compressed fiber core surrounded by a stretched shell, the fiber will naturally have a prestrain. Our data indicate that this initial strain is a necessary precursor to successful lysis. The continuum mechanics model is incomplete in that it does not predict a mechanism by which the unstrained core can be lysed, but it does show how the lysis of the outer protofibrils of a fiber can lead to elongation. This model could be extended to include plasmin kinetics and realistic molecular binding sites and accurate molecular packing geometries within the fiber rather than modeling the fiber as a simple cylinder. A complete model will encompass a combination of our polymer physics framework and our continuum mechanics results. While both models can both independently account for the phenomenon of elongation, the continuum mechanics results originate from the fiber sub-architecture that the polymer model tries to capture. The stage-like progression of lysis and plasmin binding as well as the physical differences between the exterior and interior of fibers can be accounted for in a combined model.

**References**

1. Helms CC, Ariens RA, Uitte de Willige S, Standeven KF, Guthold M (2012) alpha-alpha Cross-links increase fibrin fiber elasticity and stiffness. Biophys J 102: 168-175.

2. Houser JR, Hudson NE, Ping L, O'Brien ET, 3rd, Superfine R, et al. (2010) Evidence that alphaC region is origin of low modulus, high extensibility, and strain stiffening in fibrin fibers. Biophys J 99: 3038-3047.

3. Lottenberg R, Christensen U, Jackson CM, Coleman PL (1981) Assay of coagulation proteases using peptide chromogenic and fluorogenic substrates. Methods Enzymol 80 Pt C: 341-361.

4. Weisel JW (2007) Structure of fibrin: impact on clot stability. J Thromb Haemost 5 Suppl 1: 116-124.

5. Hudson NE, Houser JR, O'Brien ET, 3rd, Taylor RM, 2nd, Superfine R, et al. (2010) Stiffening of individual fibrin fibers equitably distributes strain and strengthens networks. Biophys J 98: 1632-1640.

6. Gorkun OV, Veklich YI, Medved LV, Henschen AH, Weisel JW (1994) Role of the alpha C domains of fibrin in clot formation. Biochemistry 33: 6986-6997.

7. Medved LV, Gorkun OV, Manyakov VF, Belitser VA (1985) The role of fibrinogen alpha C-domains in the fibrin assembly process. FEBS Lett 181: 109-112.

8. Tsurupa G, Hantgan RR, Burton RA, Pechik I, Tjandra N, et al. (2009) Structure, stability, and interaction of the fibrin(ogen) alphaC-domains. Biochemistry 48: 12191-12201.

9. Tsurupa G, Pechik I, Litvinov RI, Hantgan RR, Tjandra N, et al. (2012) On the mechanism of alphaC polymer formation in fibrin. Biochemistry 51: 2526-2538.

10. Liu W, Jawerth LM, Sparks EA, Falvo MR, Hantgan RR, et al. (2006) Fibrin fibers have extraordinary extensibility and elasticity. Science 313: 634.

11. Flory PJ (1950) Statistical Mechanics of Reversible Reactions of Neighboring Pairs of Substituents in Linear Polymers. J Am Chem Soc 72: 5052-5055.

12. Flory PJ, Rehner J (1943) Statistical Mechanics of Cross-linked Polymer Networks I. Rubberlike Elasticity. J Chem Phys 11: 512-520.

13. Ding ZY, Aklonis JJ, Salovey R (1991) Model filled polymers. VI. Determination of the crosslink density of polymeric beads by swelling. J Polym Sci Part B: Polym Phys 29: 1035-1038.

14. Medved L, Nieuwenhuizen W (2003) Molecular mechanisms of initiation of fibrinolysis by fibrin. Thromb Haemost 89: 409-419.
