# Supplementary figures and images for: Physical Determinants of Fibrinolysis in Single Fibrin Fibers
Source: PLoS One. 2015 Feb 25;10(2):e0116350. doi: 10.1371/journal.pone.0116350 (PMC4340865; doi:10.1371/journal.pone.0116350)

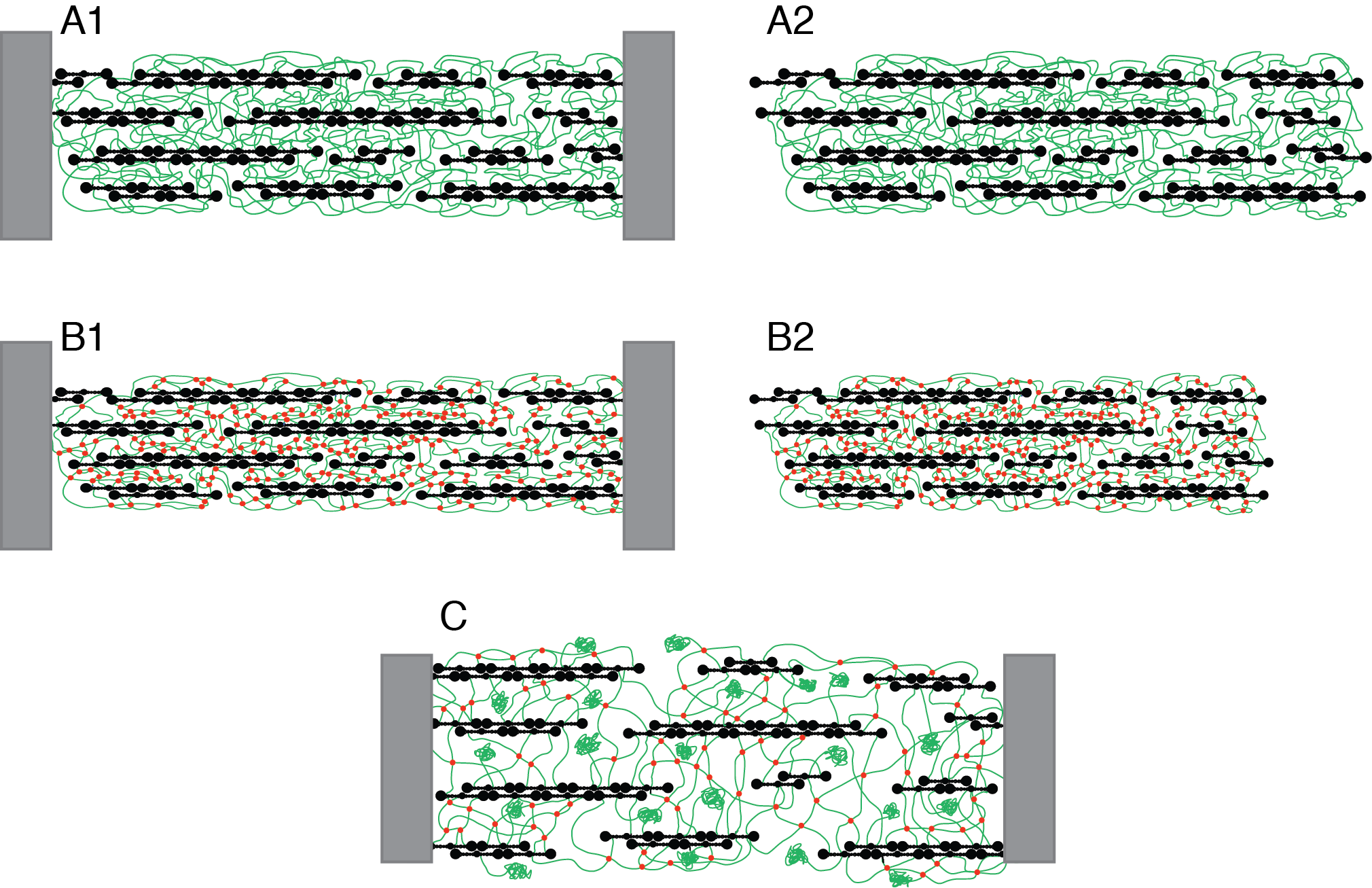

Supplement: S1 Fig — A A structural diagram for the fibrin fiber, consisting of protofibrils (black) with un-crosslinked αC regions (blue), suspended across the structured surfaces (1) and in free space (2). B As the unstructured αC regions become intercross linked (red), the network contracts, but the fiber constrained at both ends can only shrink in the diameter, not length-wise. This leads to the fiber being in a pre-strained state; the fiber is stretched beyond its equilibrium length. C The number of crosslinks is reduced during fibrinolysis in fibers with sufficient prestrain to undergo binding with fibrinolytic agents. The reduction in the number of crosslinks leads to an expansion of the network and the lysed αC regions form bundles. (TIF) [file pone.0116350.s002.tif]

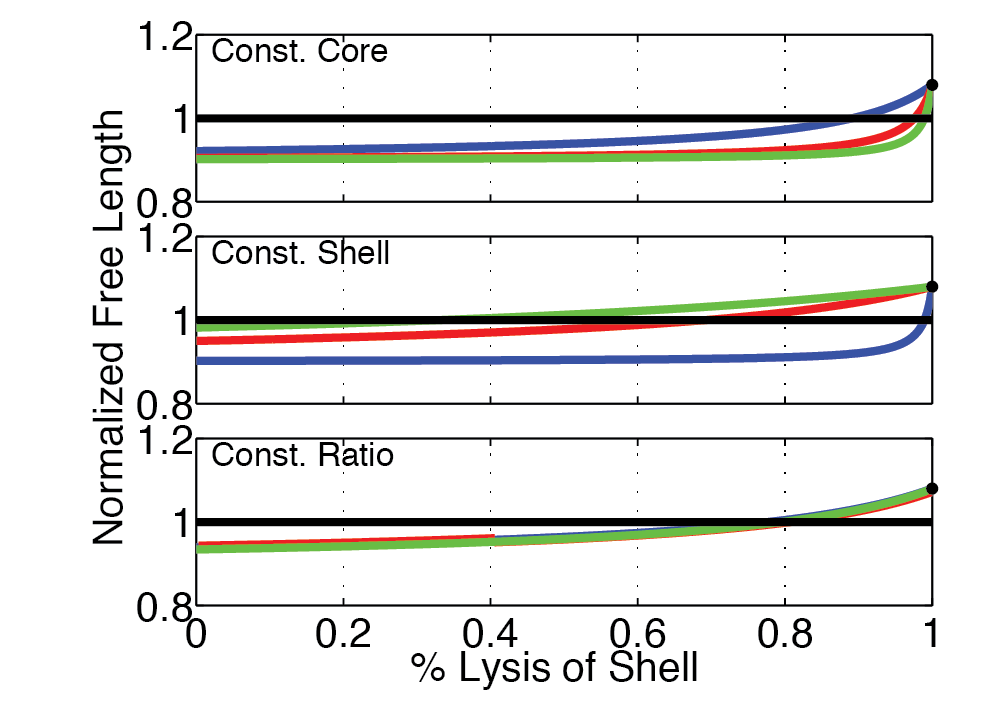

Supplement: S2 Fig — The fiber free length, normalized to the length between the structured surfaces, for thin (blue, 40 nm radius), medium (red, 80 nm radius), and thick (green, 120 nm radius) fibers as the shell is lysed. The black line signifies the SS length (LSS) and the black dot is the free length of the fiber after 100% lysis of the shell (i.e. the core length). Note that there is no radial dependence on the free length for fibers of the constant ratio model. For this plot LoA = 18 μm, S = 1.2. For the constant core, the core thickness was 15 nm; for the constant shell, the shell thickness was 35 nm; for the constant ratio, the radius of the core was half the radius of the whole fiber. (TIF) [file pone.0116350.s003.tif]
